# Cold plasma treatment for cotton seed germination improvement

**DOI:** 10.1038/s41598-018-32692-9

**Published:** 2018-09-26

**Authors:** Gerard J. J. B. de Groot, Andy Hundt, Anthony B. Murphy, Michael P. Bange, Anne Mai-Prochnow

**Affiliations:** 1 CSIRO Manufacturing, PO Box 218, Lindfield, NSW 2070 Australia; 2 CSIRO Agriculture and Food, Australian Cotton Research Institute, Narrabri, NSW 2390 Australia

## Abstract

Adverse environmental conditions at planting, such as cold temperature or water limitation, can lead to a reduced level of seed germination and plant establishment for cotton. Cold atmospheric-pressure plasma (CAP) treatment of cotton seeds prior to planting may help alleviate this problem. CAP is ionised gas that has a range of biological activities due to the formation of a mix of reactive oxygen and nitrogen species (RONS), excited molecules, charged particles and UV photons. Our results show that a 27 minutes CAP treatment using air can significantly increase water absorption of the seed, and improve warm germination, metabolic chill test germination and chilling tolerance in cotton. We also observe that the beneficial effect of CAP treatment is long-lasting and stable as improved germination activity is still seen when treatment occurs 4 months before germination testing, suggesting that future large-scale industrial seed plasma treatments may still be effectively applied well (months) before the seed planting. We conclude that CAP treatment is a promising new tool for use in the cotton industry that has the potential to significantly improve plant establishment in a wider range of environmental conditions.

## Introduction

Planted seeds require the highest probability of survival and plants need to grow as efficiently as possible to help support improved productivity to meet the increasing demands of society for both food and fibre. Germination, seed emergence and subsequent seedling vigour are critical for good establishment and healthy early plant growth. In cotton production systems, poor germination and emergence and low early seedling vigour can lead to reductions in yield^1^. In addition, the presence of pathogens, water stress and other abiotic stresses (such as temperature and salt) can affect the successful establishment of a crop.

There are currently several approaches, including treatment with chemical sanitizers^2^, organic and inorganic acids^3^, antibiotics^4^, biocontrol bacteria^5^ and hot water^6,7^, in addition to genetic modification of the seeds^8^, that support crop establishment. However, these treatments are not always efficient enough in reducing pathogens. They can also be toxic or expensive. Thus a non-toxic and low-cost alternative for efficient seed decontamination is desirable.

Plasma treatment of seeds is a new approach that is being proposed to assist germination and survival^9^. Plasma is formed by a discharge in a gas, and in the case of an air plasma consists of ions, energetic electrons, neutral species, reactive oxygen species (ROS) and reactive nitrogen species (RNS) and produces electromagnetic radiation such as UV. Preliminary investigations have confirmed that plasma pre-treatment of seeds of important agricultural crops is an effective tool for improvement of germination, shoot, and root growth^10,11^. The plasma treatments provide good fungicidal and bactericidal effects, and increased water permeability through surface coat etching and stimulation of germination and seedlings growth^11–16^. Two early studies on plasma treatment of cotton seeds exposed to a plasma glow discharge (low pressure, 3 mm Hg) observed a significant increase in early germination, but the total germination did not increase^17,18^. However, the latter study also reported that the radicle length and weight of the seed had increased significantly^17^.

More recently, it was demonstrated that plasma treatment induces desirable characteristics in the seeds through surface modification by the charged particles and neutral radicals formed in the plasma^19^. Such morphological changes to the seed surfaces are considered safe and unlikely to have any genetic impact. While the mechanisms behind improved germination of seeds after plasma treatment are not yet fully understood, a study was conducted on cotton seeds using Fourier transform infrared (FTIR) spectroscopy and emission spectroscopy to obtain details of the plasma–seed-surface interactions^19^. The study observed biologically-reactive oxygen and nitrogen species, for example, NO, N_2_O and O, in FTIR measurements of the exhaust gas and in the emission spectrum of the discharge. Such species can interact with the seed surface and partially penetrate into the seed, thereby stimulating the biochemical processes required for seed germination.

Here we show the effect of cold plasma treatment on black, acid-delinted cotton seeds. Using a laboratory-scale, non-thermal atmospheric-pressure dielectric barrier system and a range of different germination tests, we demonstrate a germination-enhancing effect of the plasma treatment under optimal (warm) and sub-optimal (cold) temperature conditions.

## Results

### Plasma equipment

**Figure 1:**
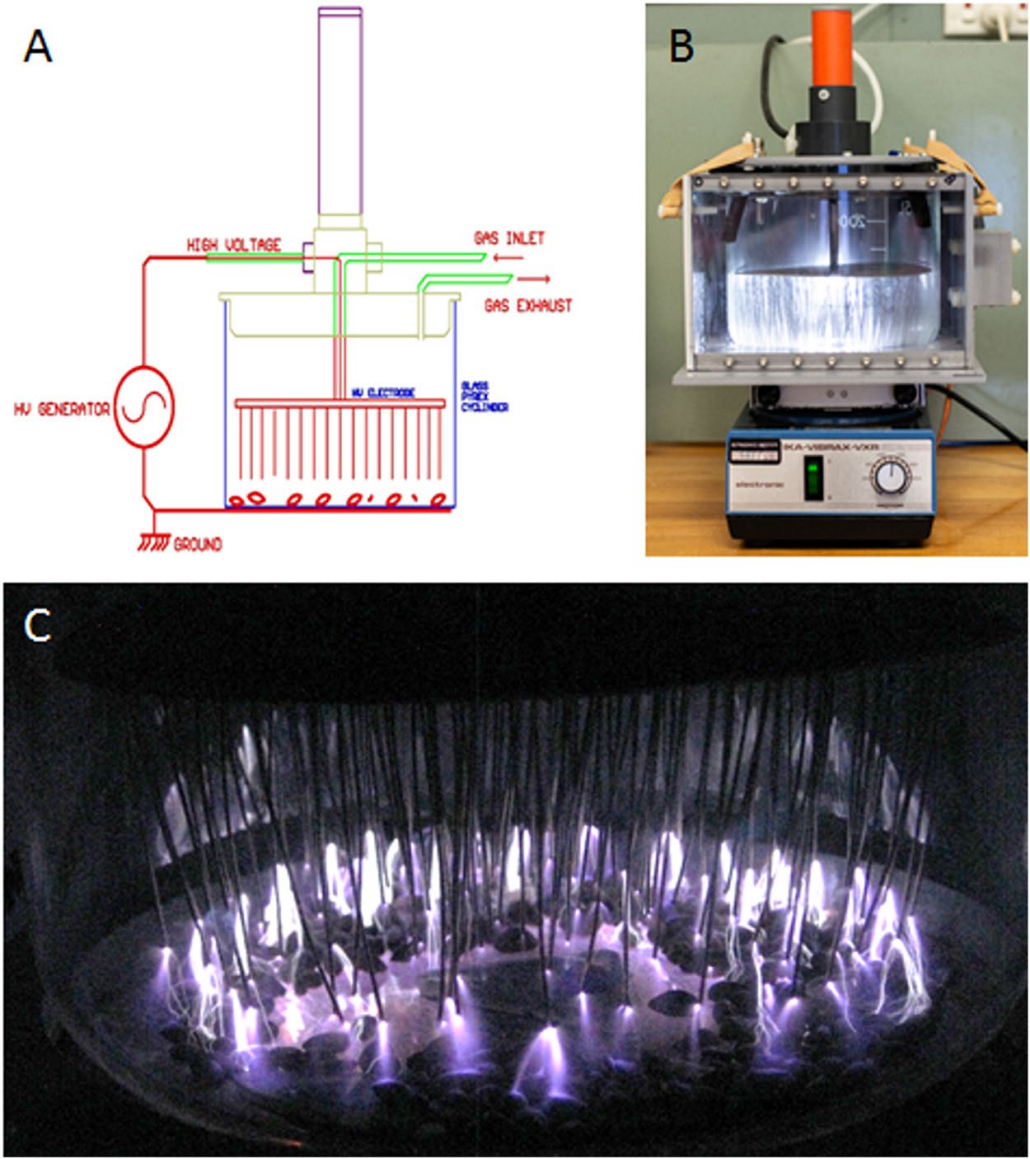
A schematic (**A**) and photograph (**B**) of the plasma dielectric-barrier discharge treatment chamber and a photograph of cotton seeds undergoing plasma treatment in the chamber (**C**).

A schematic and photograph of the plasma treatment chamber are shown in Fig. 1 and the procedure of plasma seed treatment is described in the materials and method section. Briefly, seeds were placed in a large beaker, containing an array of electrodes where the plasma is formed (Fig. 1C). A gas inlet was connected to either compressed air or argon. The temperature did not exceed 45 °C during the course of the experiments.

### Seed water absorption (hydrophobicity) measurements

**Figure 2:**
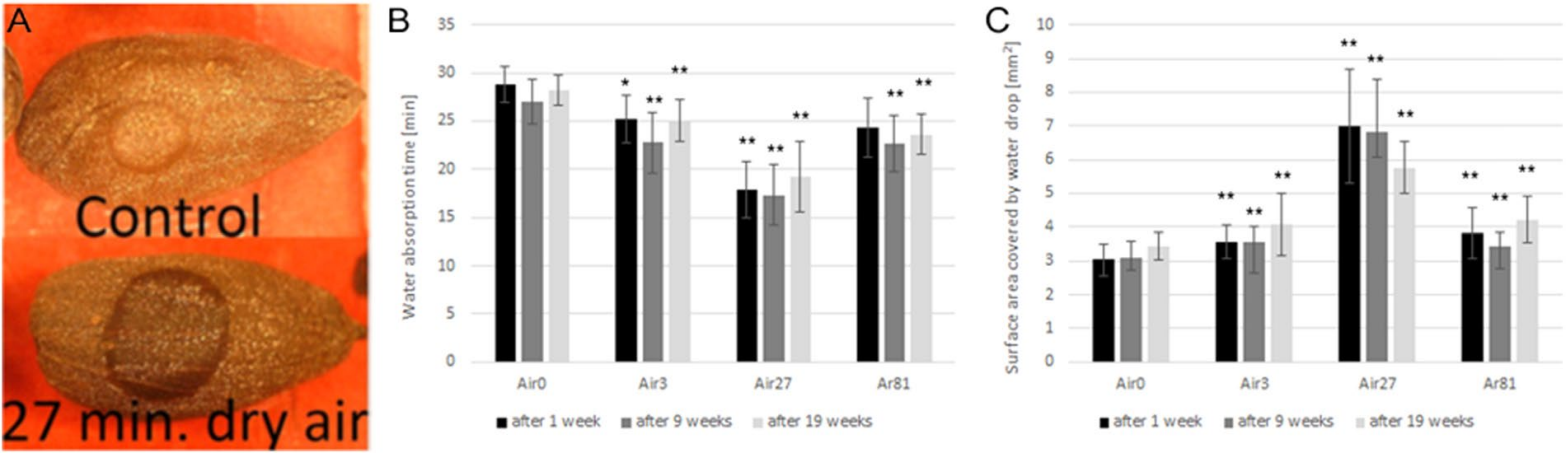
Water absorption measurements. Images of water drops on plasma-treated seeds (**A**) show the difference in the spreading of the water drops on the seeds for the control and a 27-min air plasma treated surface. Water-drop absorption times (**B**) and water-drop contact areas (**C**) were measured for control and plasma-treated cotton seeds. Error bars are the standard deviation from the mean. Student’s *t*-test calculation indicates statistically significant differences for *p < 0.1 and **p < 0.05, respectively. Air0 is control, Air3 is 3 min air, Air27 is 27 min air and Ar81 is 81 min argon.

The hydrophobicity of the plasma-treated seed surfaces was studied by measuring the water-drop absorption time and the water drop contact area on the seed (Fig. 2). Figure 2A shows that the area over which water drops are absorbed is smaller in the control than in the plasma-treated seeds, suggesting plasma treatment makes the seeds more hydrophilic and thus the water spreads out over a larger area and is absorbed faster into the seed.

The absorption measurements were performed at 1, 9 and 19 weeks. Interestingly, the absorption times and water-drop contact areas remained largely unchanged within each sample over that period. For example, all 3-min air plasma samples have similar values regardless of storage time, suggesting that the plasma treatment induces a permanent change in the surface properties of the seeds (Fig. 2B,C). All treatments resulted in a statistically significant change in water absorption time and surface area coverage compared to the control.

The 27-min air plasma treated seeds showed the largest change in absorption time (Fig. 2B) and contact area (Fig. 2C) compared to the control seeds; both changes are significant.

### Warm germination tests

**Figure 3:**
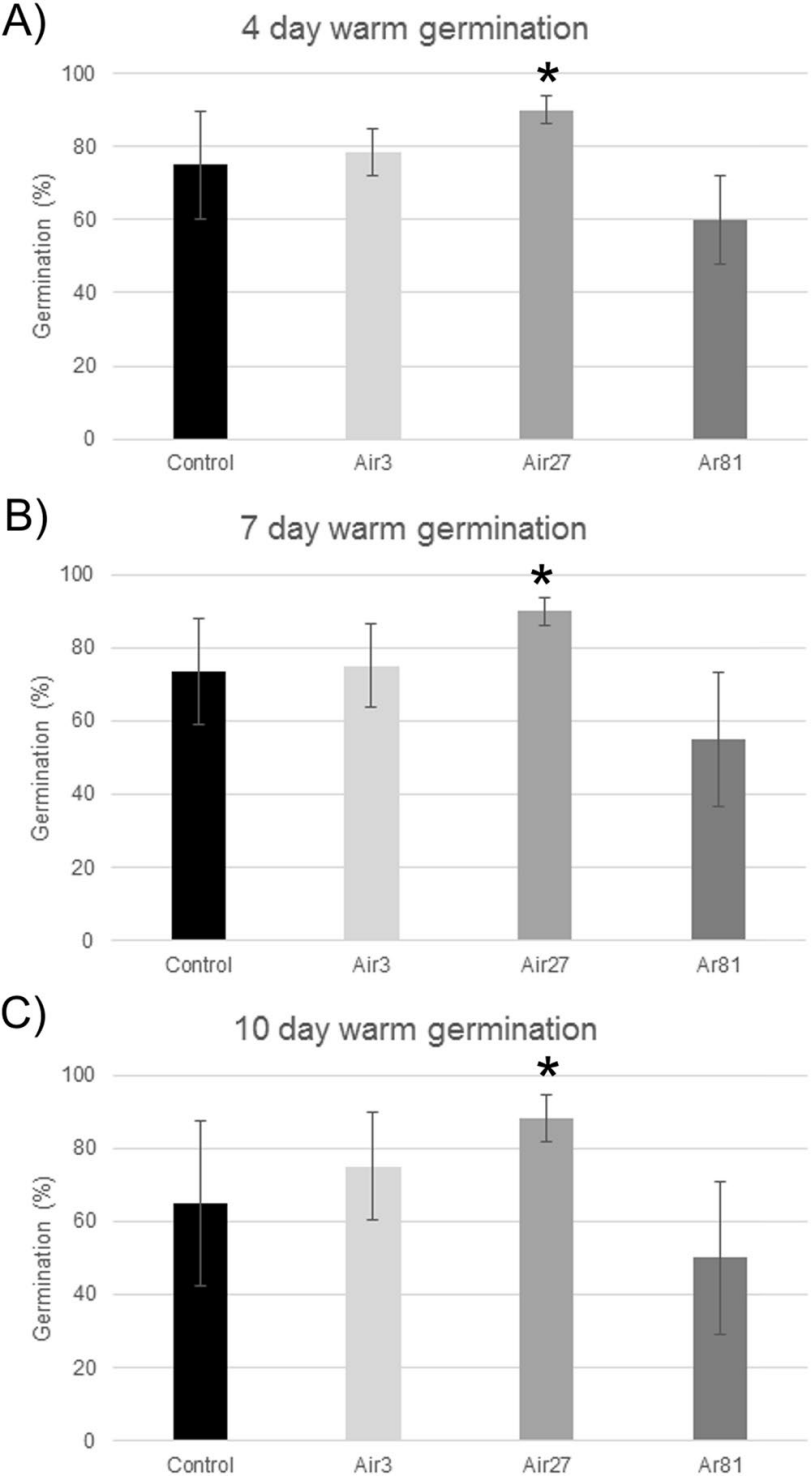
Results of warm germination studies measured at 4 (**A**), 7 (**B**) and 10 (**C**) days, respectively. Error bars are the standard deviation from the mean. Student’s *t*-test calculation indicates statistical significant difference for *p < 0.1. The seed treatments are denoted as in Fig. 2.

For the 4-day warm germination tests, germination was improved for both 3-min and 27-min air plasma treatments after 4, 7 and 10 days (Fig. 3). The argon treatment showed the lowest germination of all treatments. Across all treatments, the 27-min air treatment had the highest germination rate compared to the control for measurements at 4, 7 and 10 days and this improvement is statistically significant at the 90% confidence interval.

### Cold germination tests

**Figure 4:**
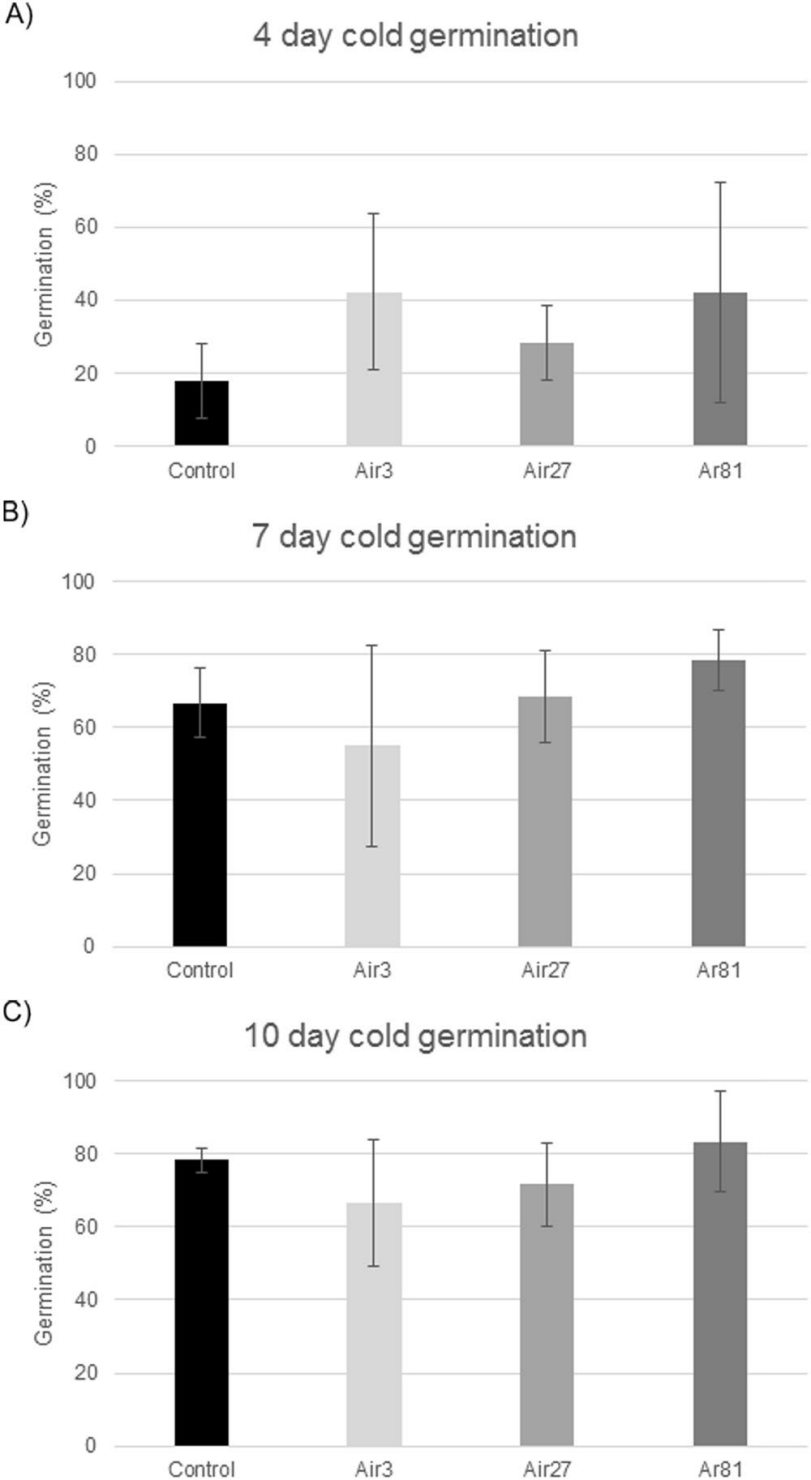
Results of cold germination studies measured at 4 (**A**), 7 (**B**) and 10 (**C**) days, respectively. Error bars are the standard deviation from the mean. The seed treatments are denoted as in Fig. 2.

While no statistically significant differences in cold germination were measured at 4, 7 or 10 days, there was a clear trend for the plasma treated seeds to have a higher germination rate than the control at 4 days germination (Fig. 4A). In fact, the cold germination rate more than doubled for 3-min air plasma and 81-min argon plasma treated seeds at 4 days germination. At 7 and 10 day germination, only the argon plasma treated seeds had a higher germination rate than the control, while the air plasma treated seeds had lower germination rates (Fig. 4B,C).

### Metabolic chill tests

**Figure 5:**
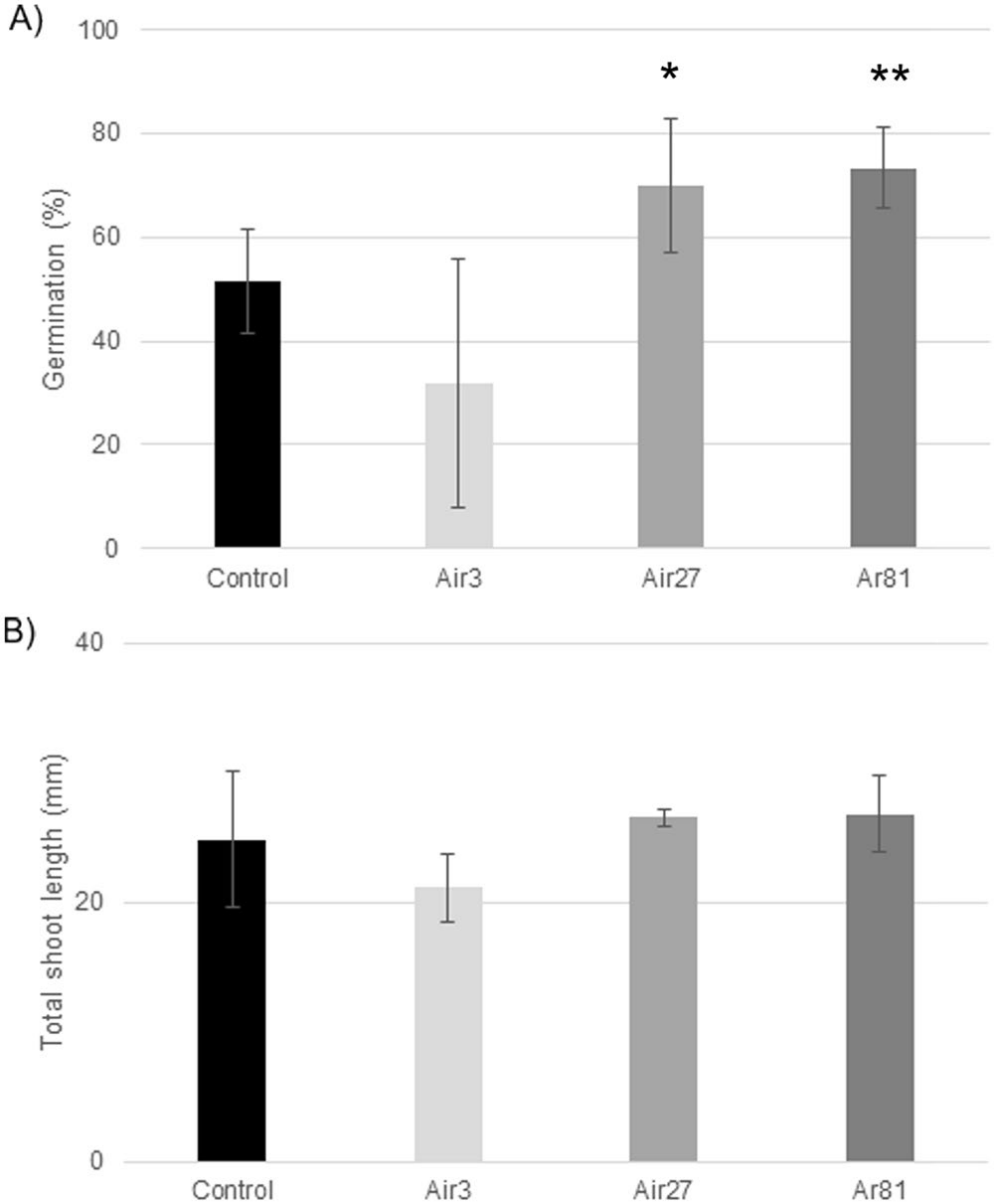
Germination results (**A**) and plant height (**B**) resulting from the metabolic chill test. Error bars are the standard deviation from the mean. Student’s *t*-test calculation indicates statistical significant difference for *p < 0.1 and **p < 0.05, respectively. The seed treatments are denoted as in Fig. 2.

Plasma treatment could improve germination rates as measured in the metabolic chill test (Fig. 5). There were statistically significant differences in germination rates of the 27-min air plasma (P < 0.1) and the 81-min argon plasma (P < 0.05) and the control (Fig. 5A). Interestingly, the 3 minute air plasma treatment (Air3) had a lower germination rate than the control (Air0) in this test. Although not statistically significant, the mean shoot length differences reflected the germination measurements with improvements (larger shoot length) for 27 min air and 81 min argon plasma treatment and a decrease in shoot length for 3 min air plasma (Fig. 5B).

### Imbibitional chilling tests

The imbibitional chilling tests revealed no statistically significant differences nor any other definitive trends for the plasma treatments relative to the controls. The data are presented in the Supplementary Material.

### Imbibition tests

**Figure 6:**
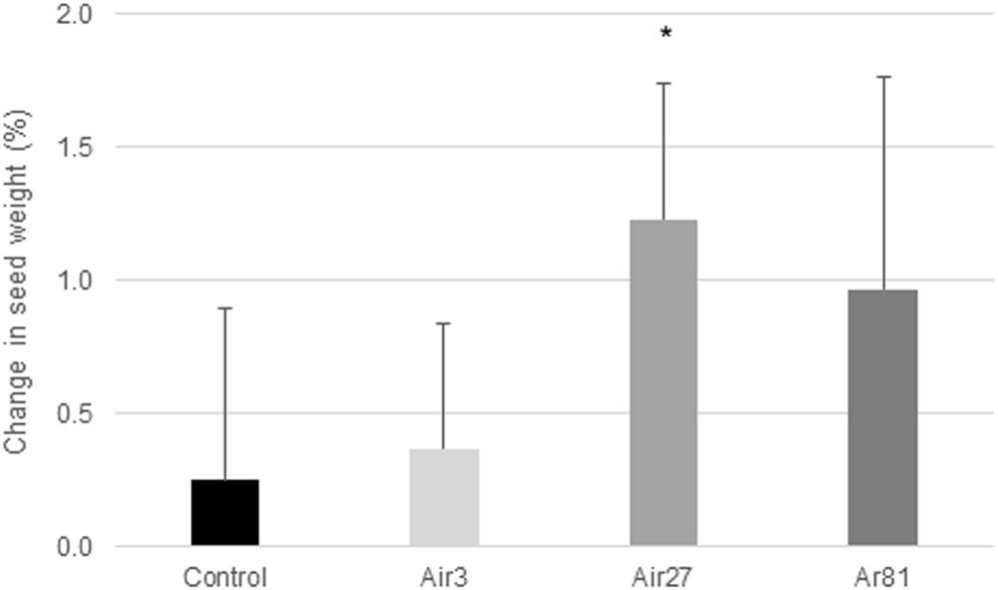
Change in seed weight as a result of the imbibition of water into the seed. Error bars are the standard deviation from the mean. Student’s *t*-test calculation indicates statistical significant difference for *p < 0.1.The seed treatments are denoted as in Fig. 2.

The imbibition of water into the seed showed that there was a trend for the plasma treatments to change the seed weight, with the weight increase lowest in 3-min air and much greater in the 27-min air and 81-min argon plasma treated seeds (Fig. 6). The higher seed weight in the 27-min air plasma sample is statistically significant (P < 0.1). Overall the imbibition test results are similar to those of the metabolic chill test.

### Electrolyte Leakage Tests

Electrical conductivity measurements indicate the degree of chilling injury. A higher measurement means a higher degree of chilling effect. However, no statistical differences in seed electrolyte leakage were measured and there were no definitive trends for the plasma treatments to give different results from the control. The data are presented in the Supplementary Material.

### Cool warm seedling length tests

**Figure 7:**
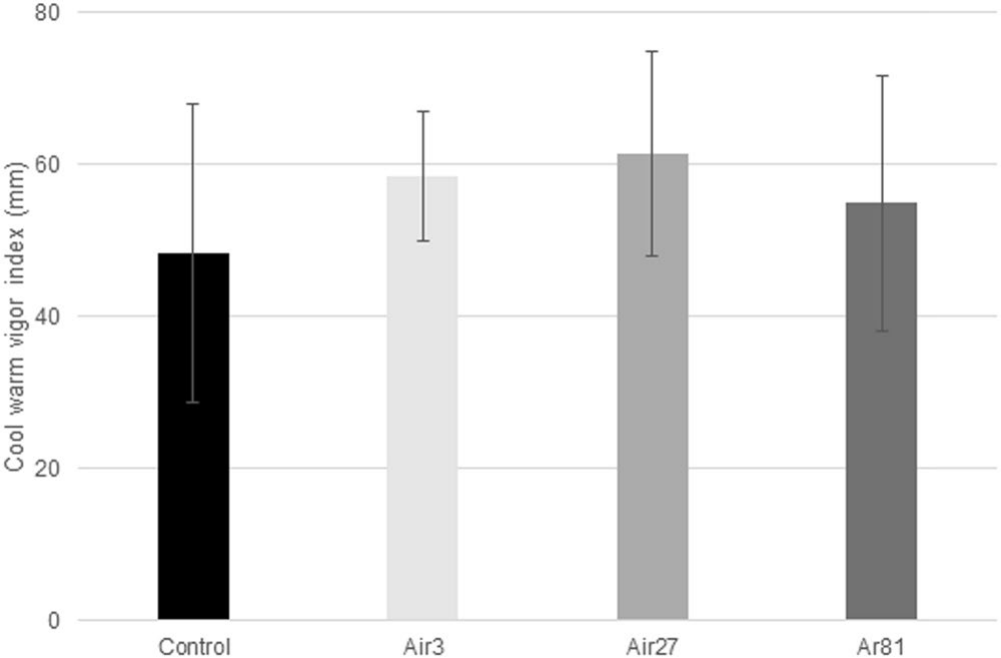
Results of the cool warm vigour index. Error bars are the standard deviation from the mean. This index is the average of the warm (30 °C) seedling length on day 4 and cool (14 °C) seedling length on day 7. The seed treatments are denoted as in Fig. 2.

No statistically significant differences in the cool warm vigour index were measured, however, there was a trend for the plasma treatments to be better than the control (Fig. 7). In particular, the mean value for the 3 minute air plasma treatment was higher than that for the control, and the mean value for the 27 minute air plasma treatment was higher again. The mean value for the 81 minute argon plasma treatment was only slightly higher than that for the control.

### Chilling tolerance assessment

**Figure 8:**
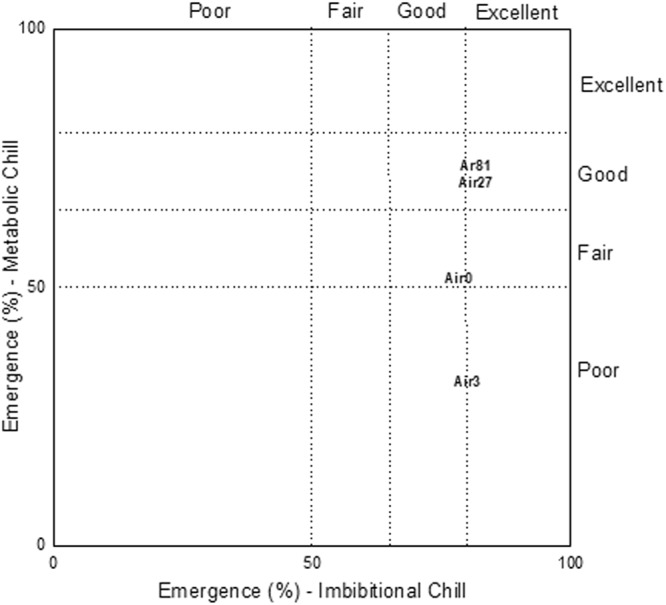
Scatter plot depicting plasma seed treatment chilling tolerances. The seed treatments are denoted as in Fig. 2.

A combined chilling tolerance assessment of the seed plasma treatments was derived from each of the two chilling tolerance tests (metabolic chill and imbibitional chill). A two-way scatter plot (Fig. 8) shows the performance of these chilling tolerance tests.

The x-axis in the graph represents the imbibitional chilling tolerance tests and the y-axis represents the metabolic chilling tolerance tests. Higher values on each axis indicate higher levels of cold tolerance with respect to that chilling test. The overall cold tolerance for a treatment is determined by its performance in both tests. Treatments with tolerance percentages from both tests of 80% or above are classified as having excellent overall cold tolerance. Those with both tolerance percentages between 65% and 80% are ranked as having good overall cold tolerance. If both tolerance percentages are between 50% and 65%, a treatment has a fair overall cold tolerance. A poor ranking is given to treatments that have either one or both tolerance percentages below 50%.

Based on these criteria the control seeds had fair cold tolerance, the 3-min air plasma treated seeds had poor tolerance, and the 27-min air and 81-min argon plasma treated seeds had similar good cold tolerance.

## Discussion

This study investigated the suitability of cold plasma treatments to improve germination success of acid-delinted black cotton seeds. Cotton seeds were exposed to a range of different cold plasma treatments using compressed air or argon gas. Both gases are inexpensive and thus suitable for possible future commercial-scale application. After treatment, seeds were subjected to several well-established germination tests that can indicate the ability of the seed to overcome adverse environmental conditions. Our data clearly demonstrate that cold plasma treatments can improve water absorption of the seeds and enhance germination rates under several environmental stress conditions. We show that water absorption and germination measures, including the four-day warm-germination and metabolic-chilling tests, and seed imbibition are significantly improved following cold plasma treatment. While other outcomes were not statistically significant, there were clear trends suggesting that the plasma treatments also positively affect cold and imbibitional chill germination and the cool warm vigour index.

Overall, the 27-min air plasma treatment showed the highest improvement in germination and water absorption tests, followed by a modest improvement for 81-min argon plasma treatment. The 3-min air plasma treatment led to a significant improvement in water absorption but showed mixed results in the germination tests. A recent study by Sivachandiran and Khacef ^20^ also showed varied results when tomato and sweet pepper seeds were exposed to a combination of direct cold plasma treatment and plasma-activated water (water that is exposed to plasma resulting in a solution with a range of active species), confirming that the choice of treatment conditions (e.g. treatment time and choice of gas) is paramount for the desired treatment outcome.

Our results show that the hydrophilicity of the seed surface increased with air plasma treatment time. It was also observed that the initial seed surface hydrophilicity changes after plasma treatment remain unchanged for several months. The latter suggests that there may be a close link between the way water drops interact with the seed’s surface and the actual germination outcomes. Similarly, Tong, *et al*.^21^ showed that plasma treatment can change water permeability of the medicinal plant seed *Andrographis paniculata* as determined by electrical conductivity measurements. A recent study showed that plasma treatment causes a reduction of trichomes on the surface of rice seeds leading to an increase in wettability which significantly affected the seed germ length but not germination^22^.

Based on the cold tolerance classification by Duesterhaus^23^, the 27-min air and 81-min argon plasma treatments led to a reclassification of the seeds from the ‘fair’ category for the control to the ‘good’ category. In general, differences in imbibitional chilling are often associated with the temperature of the water and/or the amount of seed imbibition. Higher cold water imbibition by the seeds is one cause of lower seed germination. In this study, however, plasma treatment did not affect imbibitional chilling (a chill caused by the uptake of cold water) while the cold water uptake (imbibition tests) of the 27-min air plasma treated seeds, in particular, was much higher than that of the control. The large confidence intervals of the imbibition tests did not allow further conclusions regarding a possible contradiction against usual trends with these tests. These results need further detailed investigation.

Several studies have demonstrated a favourable effect of cold plasma treatment on seed germination and plant yields of a range of important crops, including wheat^11,24^, rice^22^, barley^25^, oats^26^, soybean^27^, spinach^28^, oil seed rape^29^, green onion^30^, chick peas^31^ and cucumber^32^. For example, a study by Jiang, *et al*.^11^ showed that cold helium plasma treatment can improve seed germination, growth and yield of wheat. It was demonstrated that plasma treatment significantly improved plant height, root length and fresh weight of seedlings, and also chlorophyll, nitrogen and moisture content, indicating that cold plasma treatment could promote the growth of wheat. The mode of action of plasma-induced changes in seeds is still under debate. However, changes in the biochemical make-up of the seeds can often be measured following treatment^28,29,33^.

Our study shows that cotton seed germination is still favourably affected by plasma treatment despite a three months delay between the plasma treatment and the germination tests. This suggests that seed plasma treatments can be done on an industrial scale long before planting, hence making the need for plasma treatment on the farm immediately before planting unnecessary. Interestingly, a recent study demonstrated that positive cold plasma treatment effects can be observed for up to 17 months post treatment. It was shown that despite an initial negative effect (reduction in germination), seedling height and branching increased by up to 60% after 17 months in Norway spruce^34^.

The plasma-treated seeds have potential commercial significance in improving plant stands, reducing in-field variability, especially when conditions are unfavourable, and therefore can lead to improved yields and lessen the need to replant. Future research is needed to confirm the outcomes of this study, refine plasma treatment times (between 3- and 27-min air treatment), investigate the interaction with commercially available seed coating treatments, assess other commercial cultivars and undertake field assessments.

## Materials and Methods

### Plasma equipment

A schematic and photograph of the plasma treatment chamber are shown in Fig. 1. The chamber is a 5 L borosilicate glass cylinder that is suitable to treat approximately 350 cotton seeds at a time. The chamber is covered with four smooth triangular shaped inserts to promote seeds moving in and out of the centre; with one insert in the centre with sides approximately 41 mm long and three inserts around the outside with sides approximately 29 mm long. The chamber is placed on a shaker (IKA-VRAX-VXR) operated at 120 rpm to cause seed rolling and mixing, ensuring uniform treatment. The plasma generator was operated with air or argon gas at a flow rate of 1 L min^−1^. The high voltage electrode was connected to an AC power supply (TREK Inc., model 20/20C) using a 1 kHz sine wave with 38 kVpp for air and 11 kVpp for argon. To provide maximum exposure of the seeds to the plasma, the electrode consists of 100 needles that can be lowered into the chamber (Fig. 1). Upon operation, plasma forms around each of the needles. The distance between the needle tips and the bottom of the glass cylinder is approximately 13 mm.

### Cotton seeds

Acid delinted, black seed of the commercial cotton variety Sicot 74BRF was obtained from Cotton Seed Distributors Ltd.

### Plasma treatment of cotton seeds

Following preliminary investigations in which changes in seed surface properties were observed (data not shown), the following treatment conditions were chosen to cover a range of seed surface properties: Air0–control seeds, Air3–3 minutes dry air, Air27–27 minutes dry air, and Ar81–81 minutes argon. Approximately 350 seeds were placed into the reactor for each treatment. The temperature was monitored during the treatments using an infrared non-contact thermometer (Dick Smith Electronics, Australia). After treatment, seeds were stored in closed glass jars at room temperature before being tested for water absorption and germination.

### Water absorption test

Measuring the ability of seeds to absorb water is a simple indicator of seed surface properties, with a fast absorption time and a large water contact area resulting from high hydrophilicity. To determine the hydrophilicity of the seeds before and after plasma treatment, 2 µL tap water drops were placed on individual seeds and the time for complete absorption was measured. In addition, the water contact area was determined after the water drop had been completely absorbed into the seed. Water absorption tests were performed at 1, 9 and 19 weeks after plasma treatment.

### Germination studies

For each specific plasma treatment, approximately 1600 cotton seeds were available for germination tests. Seeds with similar size were selected for the germination tests, resulting in each test having 4 replicates of 15 seeds. Germination tests were performed three months after the plasma treatment.

For each plasma treatment described above, the seeds were subjected to the following tests:

- Warm and cold germination tests – Seeds were incubated at 30 °C (warm) and at 14 °C (cold) and germination percentage and seedling lengths were measured at 4, 7 and 10 days according to the standard rolled paper towel test^35^. The incubator was cleaned and sterilized with a 5% chlorine solution between each use.
- Metabolic chill test – Seeds were planted in sand trays (5 cm deep) and germinated at 18 °C in a growth room. After 21 days, germination percentages, and above-ground and total seedling heights were measured according to the method of Duesterhaus^23^.
- Imbibitional chilling test – Seeds were spread on a polyurethane foam pad and rolled up into a tube. The tube was filled with cool water and placed in the cold room at 5 °C for 6 hours. Seeds were then germinated in sterile trays of sand at approximately 30 °C in a glasshouse. After 14 days, germination percentages were measured.
- Imbibition test – Seeds were submerged in 5 °C water for 6 hours, air dried for 18 hours and weighed to determine weight gain percentages according to Duesterhaus^23^.
- Electrolyte leakage test – Seeds were rinsed in deionized water and then placed in 50 mL falcon tubes with 30 mL of deionized water at 5 °C and allowed to imbibe for 24 h. Conductivity measurements were then taken as previously described^23,36^.
- Cool Warm Vigour Index was calculated following the method of Tuck, *et al*.^37^. This index is the average of the warm (30 °C) seedling length on day 4 and cool (14 °C) seedling length on day 7.

### Statistical analysis

Results are presented as means of 4 replicates with standard deviation. A Student’s *t*-test (2 tailed, homoscedastic) was applied to the control and each plasma treatment individually and data is shown as significant with either p < 0.05 (95% confidence) or p < 0.1 (90% confidence).

## Electronic supplementary material

Supplementary Material

## Data Availability

The datasets generated during the current study are available from the corresponding author on reasonable request.
