# Supplementary material for: Cold plasma treatment for cotton seed germination improvement
Source: Sci Rep. 2018 Sep 26;8:14372. doi: 10.1038/s41598-018-32692-9 (PMC6158256; doi:10.1038/s41598-018-32692-9)
Supplement: Supplementary file 1 — Supplementary Material [file 41598_2018_32692_MOESM1_ESM.pdf]

Supplementary Material

Cold plasma treatment for cotton seed germination improvement

Gerard J.J.B. de Groot, Andy Hundt, Anthony B. Murphy, Michael P. Bange and Anne Mai-Prochnow

The results of the imbibitional chilling tests are given in Table S1.

Table S1. Results of imbibitional chilling tests, giving mean germination percentage for each sample, and standard deviation from the mean.

**Sample**	**Mean germination (%)**	**SD (%)**
Control	78.1	11.5
3 min air plasma	80.0	5.3
27 min air plasma	81.9	13.6
81 min argon plasma	82.3	6.3

The results of the electrolyte leakage test are given in Table S2.

Table S2. Results of electrolyte leakage test, giving mean conductivity for each sample, and standard deviation from the mean.

**Sample**	**Mean conductivity (μS)**	**SD (μS)**
Control	425	36
3 min air plasma	475	35
27 min air plasma	425	11
81 min argon plasma	458	71
